# Evolving and Improving the Sustainability of Molecular Tumor Boards: The Value and Challenges

**DOI:** 10.3390/cancers16162888

**Published:** 2024-08-20

**Authors:** Marius Bartels, Benoist Chibaudel, Rodrigo Dienstmann, Janne Lehtiö, Alberta Piccolo, Olivier Michielin, Grainne O’Kane, Giancarlo Pruneri

**Affiliations:** 1 Department of Medical Oncology, Praxis für Onkologie Mönchengladbach, 41066 Mönchengladbach, Germany; 2 Department of Medical Oncology, Hôpital Franco-Britannique—Fondation Cognacq-Jay, Cancérologie ParisOuest, 92300 Levallois-Perret, France; 3 Oncology Data Science (ODysSey) Group, Vall d’Hebron Institute of Oncology, 08035 Barcelona, Spain; 4 University of Vic-Central University of Catalonia, 08500 Vic, Spain; 5 Oncoclínicas, São Paulo 04543-906, Brazil; 6 Science for Life Laboratory and Karolinska Comprehensive Cancer Center, Department of Oncology and Pathology, Karolinska Institutet, 17177 Solna, Sweden; 7 Pathology Unit 2, Department of Diagnostic Innovation, Fondazione IRCCS Istituto Nazionale dei Tumori, 20133 Milan, Italy; 8 Department of Oncology and the Precision Oncology Service, Geneva University Hospital, 1205 Geneva, Switzerland; 9 Trinity St. James’s Cancer Institute, St. James’s Hospital, D08 NHY1 Dublin, Ireland; 10 Department of Oncology and Hematology-Oncology, University of Milan, 20122 Milan, Italy

**Keywords:** molecular tumor board, sustainability, precision oncology, molecularly guided therapy, recommendations

## Abstract

**Simple Summary:**

As the complexity of medical data and the specificity of cancer treatments increase, the role of Molecular Tumor Boards (MTBs) becomes ever more crucial. These boards expertly utilize detailed patient data to tailor personalized cancer therapies, significantly enhancing treatment outcomes. However, the operational framework for MTBs lacks clarity and uniformity, which challenges their effectiveness on a global scale. To address this, in early 2023, a group of leading experts convened to establish a comprehensive framework designed to optimize MTB functionality locally, nationally, and internationally. This framework outlines ten essential elements for the success of MTBs, including advanced data management, rigorous testing protocols, and effective leadership across all levels of healthcare. Importantly, it emphasizes the necessity of integrating MTBs within both academic and community healthcare settings, as well as into national health systems, to ensure sustainable operations and widespread access to the most advanced care. This initiative promises to equip MTBs with the necessary tools to adapt and thrive in diverse health systems, ensuring that every patient receives the best possible care based on cutting-edge medical data.

**Abstract:**

The increasing volume of information for cancer care, and the evolution of molecularly guided therapies, have increased the need for molecular tumor boards (MTBs), which can integrate such data into personalized treatment plans to improve patient outcomes. However, recommendations for improving the sustainability of MTBs are lacking. A diverse committee of MTB experts was assembled (February–March 2023), with extensive experience in sustainability in healthcare ecosystems. The aim was to identify MTB-related hurdles throughout the patient journey and develop a general framework for MTBs to operate on larger scales locally, nationally, and internationally. The committee identified ten key pillars for sustainable and scalable MTBs, including technical solutions for data integration and visualization, interoperability, learning loops, clinical trial access, legal considerations, criteria for patient testing, decision standardization, making MTBs official bodies for treatment decisions, local leaders, and international networks. The need for scalable frameworks at academic and community levels was recognized, along with integrating MTBs into national health systems to enhance sustainability and ensure optimal treatment decisions. Irrespective of the health ecosystem, the sustainability and scalability of MTBs are essential. Our framework provides guidelines to address this and to help MTBs evolve towards integrated, essential components of the oncology healthcare system.

## 1. Introduction

The volume of information available for guiding cancer care has expanded rapidly in recent years due to the steadily increasing broad application of next-generation sequencing (NGS) performed on tissues, blood samples, and cells, the use of digital pathology, and of computational radiology, coupled with the use of artificial intelligence (AI) algorithms for data analysis. NGS-based comprehensive genomic profiling (CGP) is an integral part of precision oncology, in which molecularly guided therapies can be selected using tumor-specific genomic information (insertions and deletions, gene rearrangements, copy number alterations, substitutions, and genomic signatures) [1]. In addition, with the approval of tumor-agnostic indications, cancer therapy is evolving from an organ-specific approach to therapies that can treat multiple tumor types that share the same molecular alterations [2,3,4,5,6,7,8]. Integrating and interpreting the complex datasets required for evaluating which patients would most benefit from molecularly guided therapies remains a challenge.

**Figure 1:**
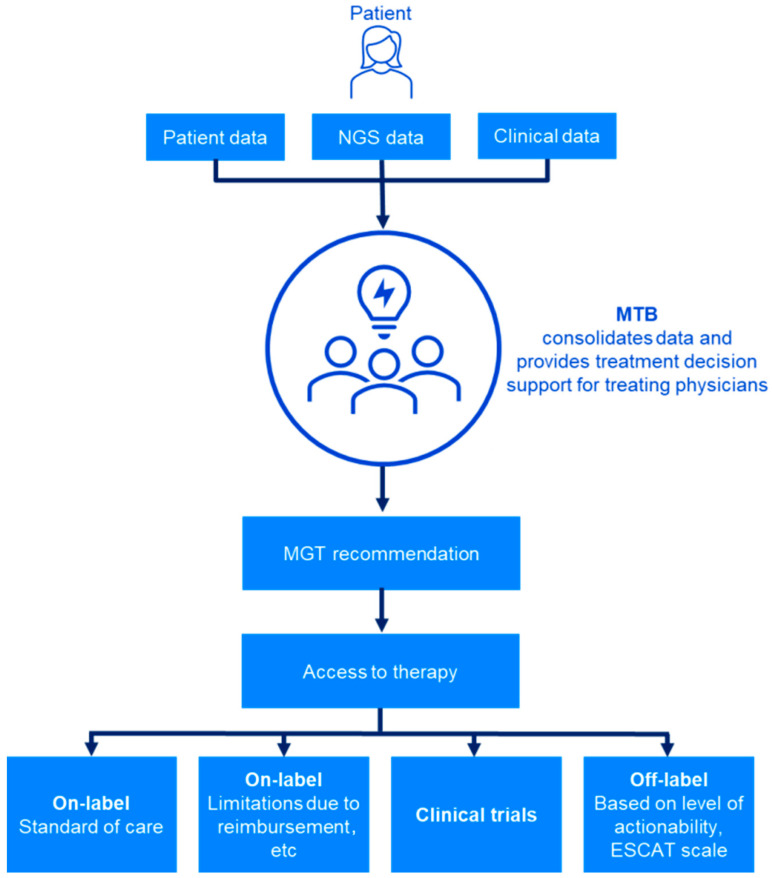
The involvement of MTBs in the therapeutic decision making process. European Society for Medical Oncology, ESMO; ESCAT, Scale for Clinical Actionability of Molecular Targets; MTB, molecular tumor board; NGS, next-generation sequencing.

Traditionally, multidisciplinary teams meet to discuss and develop a treatment plan for patients with cancer based on their tumor type; however, these teams may be underskilled in interpreting and integrating the biologic relevance and clinical actionability of genomic and transcriptomic data. This has led to the establishment of molecular tumor boards (MTBs), in which multidisciplinary experts can integrate molecular data and available treatment options into a personalized therapeutic plan for the patient to ensure the best possible outcome (Figure 1). This is particularly important for patients who present with complex genomic profiles (e.g., variants without OncoKB level 1 evidence [9] or the equivalent in the European Society for Medical Oncology [ESMO] Scale for Clinical Actionability of Molecular Targets [ESCAT] [10,11]) or who are unresponsive to existing therapy options [12].

MTBs determine patient eligibility for off-label treatments or participation in clinical trials that involve innovative molecularly guided interventions. Treatment decisions based on MTB advice have led to improved response rates and the prolonged progression-free survival of patients [13]. Further, several studies have demonstrated that MTBs significantly improve efficacy in the management of recurrent and highly resistant tumors, which are characterized by complex genetic diversity. MTBs enhance patient outcomes by facilitating personalized treatment strategies based on comprehensive genomic analyses [14]. This approach allows for targeted interventions that are specifically tailored to the genetic profile of each tumor, markedly improving treatment responsiveness and patient survival rates across different tumor types, particularly those of orphan on-label personalized treatments, with high mutational burdens or primary and secondary resistance to standard treatment [15].

The different success rates across cancer types are linked to the increasing availability of personalized cancer therapies, proved in clinical trials to benefit patients with tumors bearing multiple molecular targets and a high mutational tumor load. This complexity often presents challenging decisions for oncologists, especially when patients’ tumors exhibit multiple actionable mutations. In this scenario, MTBs play a crucial role by aiding oncologists in selecting the most efficacious treatment options. This reflects a crucial shift towards data-driven, patient-centered care, emphasizing the need for the continuous adaptation and integration of molecular diagnostics in clinical settings [15,16].

However, barriers remain for patients both in the academic setting (centers of excellence with regard to precision oncology, who may still have issues integrating multimodal data in a scalable and timely manner) and non-academic medical centers operating outside of the main academic or research institutions (where access to CGP, genomics knowledge, and clinical trials may be limited). Guidelines and recommendations for MTBs in comprehensive cancer centers and non-academic medical center settings are largely lacking. For instance, due to regulatory restrictions and insurance coverage limitations, accessing off-label therapies potentially beneficial for patients represents a challenge. Therefore, a robust decision making process including MTBs is a critical need for ensuring that patients receive the most effective and personalized treatments available [17,18].

Hence, there is an urgency to integrate MTBs into value assessment frameworks and reimbursement mechanisms for precision oncology [19,20]. Therefore, the long-term success of MTBs relies on the sustainability and scalability of well-defined processes across diverse healthcare systems to ensure that patients have equal access to the best possible treatment decisions. The continued development of the MTB concept also requires the improved integration of current use and future development of precision medicine diagnostics and clinical decisions, as well as the much wider inclusion of expertise within MTBs and the formation of a sustainable network of experts alongside the core MTB members developing diagnostics, computational tools, and analysis, with a focus on treatment decisions.

**Figure 2:**
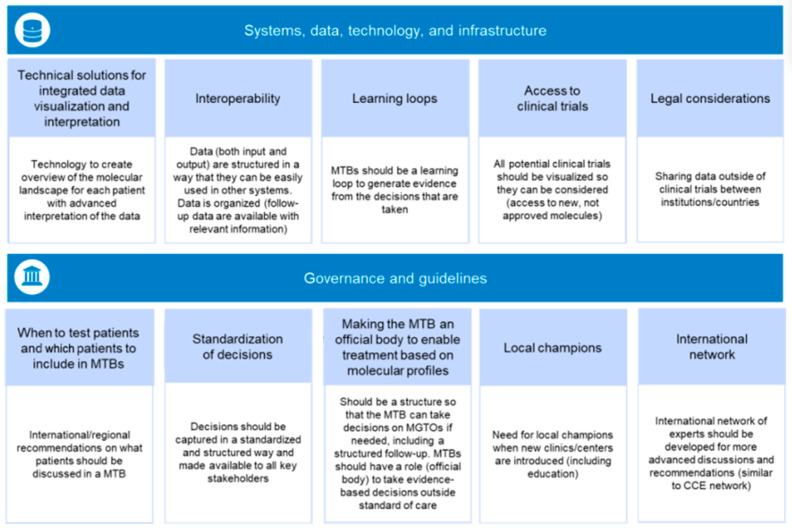
Key pillars for the development of sustainable and scalable MTBs. CCE, Cancer Core Europe; MGTO, molecularly guided treatment options; MTB, molecular tumor board.

Between February and March 2023, we assembled a diverse committee of experts with extensive experience of working with the MTB concept and aspects of sustainability in healthcare ecosystems. The committee examined MTBs in the context of different healthcare system structures (academic vs. community) based on members’ individual geographic locations, their areas of expertise (bioinformatician, scientist, oncologist, pathologist, etc.), and their specific experience with MTBs. The aim of the committee was to identify hurdles related to the implementation of the MTB throughout the patient’s journey. Here, we describe our recommendations for the development of a general framework for MTBs to expand and operate on larger scales locally, nationally, and internationally. We identified 10 key pillars across two different focus areas that are critical for sustainable and scalable MTBs from a healthcare perspective, and that can facilitate equal access to the best possible treatment decisions for all patients (Figure 2).

## 2. Results

### 2.1. Systems, Data, Technology, and Infrastructure

#### 2.1.1. Technical Solutions for Integrated Data Visualization and Interpretation

***Definition:*** The ability to use specialized information systems, programs, or platforms to integrate data, connect to up-to-date knowledge bases, and harmonize data interpretation, and to use this information to show available treatment options. Furthermore, the integration of Artificial Intelligence (AI) and Machine Learning (ML) technologies into MTBs may represent a transformative advancement in personalizing cancer treatment.

***Current situation and challenges:*** MTBs would greatly benefit from computational tools and systems that would allow, for example, the visualization of the molecular landscape for each patient and similar cohorts, with advanced interpretation of the data using up-to-date knowledge to support decision making in an interactive and dynamic manner. The data needed for an MTB to reach clinical decisions are scattered throughout different systems, often hospital specific, using various models for storage and different terminologies. However, technologic infrastructures for computer-enhanced MTBs are still immature and the complexity of existing solutions represents a burden for healthcare professionals (HCPs) and technology experts. Commercial solutions (e.g., Navify^®^, Roche Molecular Systems, Inc., Santa Clara, CA, USA) and other solutions that are linked to a particular test provider are available, as well as academically developed solutions (e.g., Cancer Core Europe’s Molecular Tumor Board Portal [21] and Hartwick’s tool). However, very few custom solutions are integrated into the local information technology environment of the hospital and clinics. This means that data from clinico-pathologic, imaging, standard biomarker, and genomics tests usually have to be uploaded manually to the MTB platform. Moreover, many clinical decision-support systems used by MTBs focus on only one data type and provide knowledge on each specific gene variant separately; MTB teams need to consider multiple diagnostic data types, as well as patient characteristics and the disease context, to reach treatment recommendations or suggest trial inclusion. Virtually no tools exist for multimodal data interpretation to support MTBs. Finally, there are few semantically tagged databases of biomarker-guided clinical trials and most MTB platforms do not present locoregional clinical trial opportunities.

***Recommendations:*** An appropriate system would be a user-friendly and interactive dynamic report, customizable to local health systems and adaptable to both academic and community hospital settings. The system would have the ability to summarize information for individual patient- and cohort-based analyses to support the use of rules-based or machine-learning approaches for therapy matching, as well as data management for follow-up and quality monitoring. Incorporating Artificial Intelligence (AI) and Machine Learning (ML) significantly enhances these capabilities. AI algorithms, for instance, can perform predictive analytics to analyze vast amounts of genomic and clinical data, identifying patterns and predicting treatment outcomes reviewing the most up-to-date information relevant to specific patient cases. This could lead to higher precision in tailoring treatment plan decisions, creating a real decision support system (DSS) [22]. Additionally, ML models facilitate real-time data processing from ongoing treatments, providing timely updates on patient responses, which is crucial for adjusting treatment plans effectively [23]. Future developments would require a collaborative approach between industry and both the medical and technology expert teams to provide the best practices, as well as lawyers for navigating data sharing and privacy in this setting (as is further described in Section 2.1.5). Systems should allow the development of multimodal diagnostics, combining genomics with imaging and other emerging ‘-omics’ data. Interoperability standards should be developed between data curation, data annotation, and clinical decision-support systems, to allow harmonization and support precision medicine studies and trials in multicenter settings (further described in Section 2.1.2). Systems should support education for multidisciplinary teams in precision medicine utilizing AI-driven tools for raw genomic data interpretation, quickly interrogate updated published data, and obtain information regarding actively recruiting clinical trials, thereby keeping MTB members updated with the most relevant information for specific patient cases [24].

#### 2.1.2. Interoperability

***Definition:*** the ability of the different systems, devices, and applications to exchange, integrate, and use data safely and effectively, as well as to generate interoperable data repositories.

***Current situation and challenges:*** There are currently no defined structures for collecting the data required for informing MTB discussions or for dealing with differences in data input. For example, genomics data could be from targeted tumor NGS alone, tumor plus germline testing, or whole exome sequencing. Sample processing could also be different (fresh tissues, formalin-fixed paraffin-embedded samples, or liquid biopsies). The reporting of patient characteristics may differ to the extent that relevant risk factors (e.g., smoking or family history of cancer) may not be included for some cases. There is also a lack of standard data and file formats, computational and security standards, nomenclature (including how variants are annotated), guidelines, and actionability thresholds for emerging biomarkers. Finally, there are currently no predefined data elements or vocabularies for collecting information on MTB decisions and subsequent patient outcomes. This lack of standardization makes MTBs less efficient and hinders their generation of systematic evidence.

***Recommendations:*** To solve this, patient data and outcomes following MTB advice should be structured in a way that can be used easily by different systems across institutions. This includes allowing retrospective data entry so that information can be identified based upon available data for relevant insights. Nomenclature should be harmonized based on existing guidelines to ensure information flow within and between MTBs. Stakeholders should actively work towards the standardization of data and file formats for different data types in national and international bodies to facilitate data analysis and the development of interpretation algorithms, and to ensure the compatibility of tools. Until automated approaches to extract data from medical records are fully operational, manual curators will need to interact with the medical team to collect relevant information that will be used in MTBs for individualized therapeutic decisions. Data should be aligned pre-MTB (e.g., type and time of biopsy, performance status, previous treatment exposure, current disease status), during the MTB (e.g., prior genomic profiling results, theoretical best treatment matches for the patient), and post-MTB (e.g., treatment decisions, patient preference, impact on HCPs’ medical practice) with well-defined and harmonized minimal dataset components, time points for data capture (e.g., 6 months after MTB discussion), and nomenclature. The definitions for each of these may be informed via surveys to MTB users or through the outcomes from clinical trials that use MTB infrastructures (e.g., WAYFIND-R [25] or the TARGET trial [26]).

#### 2.1.3. Learning Loops

***Definition:* Learning loops can be defined by the following aspects. Firstly,** the ability to use MTB experiences and decisions to improve HCP education and to generate evidence for value assessment, as well as systematizing and connecting diagnostics data to real-world outcomes in knowledge bases for efficacy, safety, and quality monitoring. Secondly, the development of a model, where treatment outcomes (successful or not) of cases with rare genomic alterations can be identified; this real-world data could then be used to guide therapy for patients with similar alterations and to refine biomarker clinical decision thresholds. Thirdly, the ability to reassess and adjust future recommendations after the incorporation of follow-up clinical data, and to develop feedback mechanisms to the wider research community to catalyze translational research, the development of computational tools, biomarker analysis, and the promotion of further clinical research.

***Current situation and challenges:*** MTBs can facilitate professional learning loops both to advance decision making and to generate evidence to develop systems, biomarkers, and treatments for future patients. Several large-scale, genomic-medicine efforts have been launched in oncology in recent years aiming to systematize learning loops. Similarly, efforts to systematize learning loops in imaging are also under way. Moreover, multiple European countries have initiated national drug repurposing trials, and an EU project (PRIME-ROSE [27]) has been set up to connect such trials, with the potential to build improved learning loops. However, sustainable learning loops connecting multimodal, biomarker-data-based clinical decisions with outcomes are lacking. Many MTB tools and existing quality registers lack the granularity of diagnostics data, which would allow the refinement of biomarker thresholds and treatment decisions. The lack of sufficient feedback loops prevents the early detection of favorable or unfavorable biomarker combinations in relation to outcomes. The rapid development of treatment repertoires and predictive biomarkers requires professional life-long learning loops for the dissemination of precision-medicine knowledge and best practices.

***Recommendations:*** All patients that are discussed in MTBs need to be followed up in a standardized way, with outcomes collected at similar time points across all patients and follow-up information connected to diagnostics data and MTB recommendations. After a patient has received an MTB-recommended treatment, a documented follow-up should be carried out every 3–6 months and the clinical outcome reported back to MTBs to help identify the most beneficial, molecularly guided, off-label treatment options. MTBs should serve as repositories for decisions by capturing outcomes to enable the more advanced use of data within evidence generation, for biomarker development (including evidence-based thresholds), and for assessing the cost–benefit of interventions. As previously mentioned, this requires the development of interoperable solutions for data capture (see Section 2.1.2); it also requires the standardization of decisions (discussed more in Section 2.2.2). The continued development of MTBs, precision medicine, and related diagnostics requires the integration of multidisciplinary teams including a wide scope of HCPs involved in generating and interpreting diagnostics data and interacting with patients. Importantly, feedback loops need to reach beyond current hospital-centered HCP teams. Rapid development in precision medicine requires feedback loops to include and feed experiences and data to experts covering multiple aspects, such as raw data handling, interpretation, and clinical decision support tools, as well as incorporating computation and diagnostics tools, methods developers, and experts in cancer biology and omics. Finally, the formation of such loops should include educational aspects, capturing knowledge gaps, misinterpretation, and the lack of action despite favorable biomarker indication. This would help to guide the sustainable development of MTBs as well as future implementation research and digital tool development.

#### 2.1.4. Access to Clinical Trials

***Definition:*** the ability to search for a comprehensive list of available clinical trials and for patients to access CGP, the relevant treatments, and the sites where clinical trials are taking place.

***Current situation and challenges:*** A substantial number of alterations identified through CGP are actionable through clinical trials only [14], which may lower the value of genomic-profiling tests and restrict the applicability of CGP in community centers with limited access to clinical trials, eventually causing inequality with regard to access to precision oncology. Pragmatic clinical trials (e.g., the Drug Rediscovery Protocol) to expand access to anticancer drugs and follow the outcomes in patients with advanced cancer who have exhausted their treatment options are underway [28]. Trials of drugs targeting rare alterations may be slow due to a lack of effective coordination, and the communication of inclusion criteria (or updates to inclusion criteria) may not be adequately communicated to HCPs, particularly for Phase I drug trials. Matching patients to clinical trials can also be hindered by the absence of standardized and up-to-date software or clinical trial registry websites searchable by MTBs; the small number of laboratories able to perform CGP, resulting in limited access to genomic tests; the use of small targeted panels that may not detect all potentially actionable targets or may not provide information on genomic signatures and possible resistance mechanisms; and the lack of availability of clinical trials that cover the broad range of potential genome-directed treatments. Additionally, patients that could be matched to clinical trials may not be able to participate for logistical reasons (e.g., unable to travel due to financial limitations).

***Recommendations:*** Access to clinical trials could be improved through the creation of a comprehensive, automated, and secure platform aimed at allowing MTBs to match patients to clinical trials, along with complementary digital solutions for patients and HCPs (e.g., mobile applications). This needs improvement via structured machine-readable updated databases with trial information. This would leverage the MTB evaluation of patients with actionable biomarkers/alterations but no on-label drugs available. It would also enable the development of national and international MTB networks which can work with health authorities to improve the reach of clinical trials.

#### 2.1.5. Legal Considerations

***Definition:*** The ability to navigate the regulatory landscape that may prevent the sharing of patient data nationally and internationally, access to off-label treatments recommended by MTBs, compliance with patient communication regulations, and the involvement of other non-HCP stakeholders required for MTB development and the secondary use of data for research.

***Current situation and challenges:*** Data sharing between countries outside of clinical trial settings may be restricted by the complexity of evolving health data policies, the processes and guidelines, and the privacy and security of personal health data. These legal hurdles mean that data sharing between MTBs has become extremely challenging at the international level. In addition, these restrictions limit collaboration between cross-border experts.

***Recommendations:*** EU legislation to remove barriers to data sharing and the cross-border discussion of patients would provide an opportunity for improved knowledge sharing and, eventually, better patient outcomes. Solutions like MedCo in Switzerland, which provides encrypted and anonymized access to medical data for research purposes, and the WAYFIND-R global pan-cancer registry data-sharing and collaboration platform [25], may help to inform the development of future solutions for MTBs. Additional legislation may also address international data sharing beyond the EU and the use of off-label treatment outside of clinical trials. MTBs regularly recommend off-label treatments so legislation should be developed to support prescribing oncologists who need to implement these recommendations.

### 2.2. Governance and Guidelines

#### 2.2.1. When to Test Patients and What Patients to Include in MTBs

***Definition:*** the development of recommendations aimed at informing HCPs on the early genomic testing of patients and guidance on those that are suitable for MTB intervention and how to submit cases to MTBs.

***Current situation and challenges:*** The organization of NGS testing for patients requires several well-established preliminary steps. A tissue biopsy specimen must be obtained, followed by adequate staging procedures, resulting in a precise histopathologic diagnosis and classification into an initial stage according to the established international staging systems (e.g., Union for International Cancer Control, American Joint Committee on Cancer, etc.). With this essential information, every patient should be assessed by a conventional tumor board to discuss further diagnostics and/or treatment initiation. As the number of targetable genomic alterations in the advanced disease first- and second-line settings increases, NGS has become more widely applied to tissue biopsy specimens, and in some cases liquid biopsies, and broader NGS panels have become more cost-effective. Molecular-pathologic risk or treatment stratification with or without the use of targeted therapies is also becoming established and drug tolerability testing is becoming statutory. Patient cases are submitted to MTBs by treating physicians and multidisciplinary teams. As there are no shared recommendations on what patients should be discussed in an MTB, this has led to a heterogenous situation where MTB access depends on awareness of the platform among HCPs, although most patients with advanced disease are tested at some point and require the use of an MTB.

***Recommendations:*** Multigene NGS should be initiated for most patients with advanced cancer at an ever earlier point in their course of treatment, ideally before the initiation of first-line therapy, in order to fulfill the standard of care. This approach would help to establish sustainable treatment strategies and patient guidance, knowing all genetic risk factors and targeted treatment options as early as possible. The growing number of tumor-agnostic targets (e.g., *BRCA1/2*, *HER2*, *NTRK1/2/3*, *BRAF v600E*, *RET*, *FGFR1/2/3, IDH1*, homologous recombination deficiency, and high microsatellite instability and tumor mutational burden) in combination with targets of ongoing clinical trials (e.g., *BRAF* Class II/II and *KRAS*) justify the use of multigene NGS in most patients with a good Eastern Cooperative Oncology Group performance status (ECOG PS) and who could derive clinical benefit from a resulting therapy. Since not all countries have equal access to NGS, healthcare policies should be developed to include genomic sequencing strategies. International/regional recommendations should be developed to ensure that patients with molecular targets with no approved therapies are prioritized in MTB discussions. Patients bearing somatic genomic variants enabling on-label targeted treatments are provided with the current standard-of-care therapy by their prescribing medical oncologist. However, all patients with a mutation potentially actionable by an off-label treatment or within a clinical trial should be discussed within an MTB and receive genetic counseling if needed.

#### 2.2.2. Standardization of Decisions

***Definition:*** the development of scales and standardized nomenclature that will allow MTB decisions to be reported in a defined way.

***Current situation and challenges:*** MTB decisions are based on the experts that are present in a particular meeting and are not captured in a standardized way, making follow-up difficult.

***Recommendations:*** A framework is required to support decision making within MTBs and the standardized reporting of these decisions. This includes the development of ESCAT [10,11] grade equivalents for tumor-agnostic biomarkers, and the use of OncoKB [9] as the gold standard for the annotation of variants and standardization of language for unknown variables, e.g., the presence of a rare point mutation of unknown functionality. In 2022, the Digital Institute for Cancer Outcomes Research in Europe (DIGICORE) completed a project across 16 cancer institutes in 13 countries to define a minimum dataset and data standard in oncology, identifying 25 essential data items required for outcomes research and the monitoring of care quality [29]. A similar list could be applied to facilitate MTB discussions and capture their decisions. New standardization initiatives are urgently needed for emerging data types, such as radiomics, digital pathology, and proteomics, as well as integrative data analysis outputs. For clinically meaningful development, these standardizations and new data types are best developed as observational data layers within MTBs at academic centers. The collection of the real-world outcomes of patients who receive MTB-recommended treatments could help MTBs to learn from their decisions and facilitate future AI initiatives (e.g., the Pancreatic Cancer Action Network’s SPARK platform [30]).

#### 2.2.3. Making the MTB an Official Body to Enable Treatment Based on Molecular Profiles

***Definition:*** the ability of MTBs to generate the evidence required for off-label treatment and for this evidence to be recognized by regulatory bodies and payers to allow patient access and reimbursement for drugs.

***Current situation and challenges:*** Even for MTB-recommended treatments, the accessing and reimbursement of off-label drugs remains a challenge in some countries, and this may be associated with delays in patient care, which can adversely affect patient outcomes. Some countries have implemented processes for reimbursement of MTB-recommended therapies. For example, in Switzerland, “Article KVV71” allows the reimbursement of drug costs by compulsory health insurance companies, provided certain conditions are met, and the Swiss Patient Access Program (industry collaboration) provided patients with access to selected oncology drugs that had been rejected by health insurance providers until the end of 2022. Following this pilot study, a program has now been implemented to further enable patient access to off-label oncology drugs and this could be used to provide learnings to other countries requiring similar programs (e.g., their checklist to standardize decision making criteria). In Italy, where regional organization of the health system has resulted in disparities in biomarkers and precision medicine access, guidelines have been developed aimed at streamlining standards of care, including the need for referral to molecular biology laboratories and recommendations for the appropriate use of NGS. Due to the relatively low volume of patients who undergo NGS in Italy and limited access to new drugs, a law has been passed that requires every region to establish MTBs for patients with no therapeutic options [31]. The Italian MTB Virtual Consultation system (MTB VCS ITA Project) is a multicenter observational study aimed at defining the decision making process of MTBs. It involves several academic centers throughout the country and collects data within a shared virtual platform on patients with advanced solid tumors who have progressed after previous or standard (according to local guidelines) treatments and who no longer have access to conventional curative treatment. In France, Haute Autorité de Santé (HAS) has granted early access authorization to innovative medicines since July 2021, following an opinion from the French National Agency for Medicines and Health Products Safety (ANSM) as to their presumed efficacy and safety.

***Recommendations:*** The recognition of MTBs by regulatory agencies and payers, as official bodies that take evidence-based decisions outside standard-of-care treatments in a structured and informed way, is required for the evolution of current MTBs. Real-world evidence from MTBs should be systematically collected using standardized reports and used to develop pay-by-performance models. Guidance should be provided on how MTB decisions should be incorporated into a country’s specific health economy setting. In addition, it is important to learn from MTB decisions so that all patients have access to treatments recommended by MTBs in an equal and fast way. The Access, Consultation, Technology, and Evidence (ACTE)-MTB maturity assessment tool was developed to assess the maturity of MTBs across four different categories (access, consultation, technology, and evidence) [32], and may represent a method of validating the quality of decision making and improving standards across different MTBs.

#### 2.2.4. Local Leaders

***Definition:*** The identification and empowering of experts and patient representatives who can improve HCPs’ awareness of MTBs to increase patient access to MTB evaluation; leaders with expertise within healthcare, academia, and tools development should also be included to support the continued development of MTBs. Also, there is a need for human curation in addition to the machine integration of data for MTB discussions and for local HCP leaders for case identification and to promote local MTB initiatives, as well as for administrative support for preparing patient data, uploading data to the MTB, collecting literature to facilitate MTB discussions, and collecting information on real-world patient outcomes following MTB-recommended treatment.

***Current situation and challenges:*** Local leaders, who are part of and have extensive experience of MTBs (medical, administrative, and pragmatic) can improve the outreach of MTBs both to patients and the wider experts needed for MTB development. However, this crucial work is often underappreciated and under resourced; therefore, efforts are needed to promote wider expertise to ensure development and to advocate for local leaders to have dedicated time to execute MTB-related tasks. Efforts from medical societies are also needed to offer training resources to MTB leaders. NGS-guided care should be offered to every treatable patient with advanced disease, as is already carried out in academic centers and centers of excellence. However, most patients are treated in community, local outpatient, or resource-limited settings where there is a lack of locally accessible MTB networks to ensure NGS-guided care.

***Recommendations:*** Improved outreach could be achieved in one of two ways. It could be either a centralized approach, where a center with an MTB receives patients from other local centers (although this may be limited by the high number of cases already requiring MTB input), or a decentralized approach, where a new MTB can be created with the help of the local leader in a secondary center with the appropriate expertise to form at least the minimum group of attendees (e.g., bioinformatician, pathologist, and human geneticist) and the availability of a platform to centralize patient data (although this may be limited due to a lack of experts). Decentralized MTBs could be used to validate the most frequent and approved situations, while central MTBs could be reserved for more complex decisions (e.g., rare alterations, doubt regarding actionability/drugability, etc.). Both complementary approaches require the evolution of current algorithms. Academic centers should also connect and promote the joint development of MTB tools and guidelines, as well as ensuring that any developments are disseminated across MTBs. Local leaders are needed to make precision oncology equally available to patients who are treated in existing structures within the community setting as well as for those who are treated in academic centers. Local community leaders should also be able to offer information on relevant clinical trials, so that as many patients as possible can participate in these. In the case of resource-limited settings we propose to improve the training of local healthcare providers in the fundamentals of genomics and precision medicine, focusing on the development of basic but robust digital infrastructures that support telemedicine and virtual tumor boards. This approach allows experts from better-equipped regions to assist in local decision making. This can be facilitated through partnerships with academic institutions and international healthcare organizations, leveraging cost-effective genomic technologies and developing open-source or low-cost clinical decision support systems tailored to specific local needs. Moreover, developing clinical guidelines specifically designed for resource-limited settings helps standardize care and make the best use of limited resources.

#### 2.2.5. International Network

***Definition:*** the development of a network of experts, both nationally and internationally, with systems that can operate across borders to allow for the exchange of knowledge and to catalyze the standardization and harmonization of recommendations.

***Current situation and challenges:*** Multiple European networks exist, which can catalyze MTB development and the standardization of best practices. The Organisation of European Cancer Institutes (OECI) provides a framework with criteria for the development of standards and harmonization of MTBs. The OECI has also initiated formalized networks of cancer centers, with the Oncologic Network Southeast Netherlands being the first such hospital network. Further, broad international networks such as the ESMO Designated Centres of Integrated Oncology and Palliative Care can play a major role in setting standards for MTBs. Defined research-intensive smaller networks, such as Cancer Core Europe (CCE), can develop and catalyze the early implementation of more ambitious MTB practices for hospitals heavily involved in clinical trials. CCE is an example of an existing multicenter weekly MTB within a structured network developing joint MTB tools and data sharing, where international experts can meet regularly. The European University Hospital Alliance (EUHA) forms a similar network with a broader focus beyond cancer. Despite these types of networks involving a limited number of academic centers, they can play an important role in piloting more ambitious practices suitable for digital tools’ development and research to evolve MTBs. Moreover, national cancer hospital networks, such as the German Hospital Federation and the French Paris Saclay Cancer Cluster (PSCC), in collaboration with agencies such as the French Society of Predictive and Personalized Medicine, can co-develop the harmonized standards for MTBs considering traits of individual national healthcare systems. There is a need to develop international standards for the wider MTB concept and make use of these networks to allow the sustainable development of standards and harmonization. Such standards are important for the development and wide use of support tools, particularly since the volume of information to be interpreted is likely to increase as more multimodal diagnostics are introduced. The fast development of new diagnostic data modalities and integration of data types calls for larger collaborative networks for harmonization and standardization to support clinical decision recommendations.

***Recommendations:*** All patients should have the opportunity to access an MTB where a network of international experts can be consulted if needed. Interconnected repositories across different centers and countries of patient cases and MTB recommendations should be established to support decision making and reduce the burden on networks. Precision oncology relies heavily on the availability of big data. Larger datasets on MTB recommendations and the corresponding patient outcomes mean that MTBs can work more empirically. Therefore, an international network that allows the exchange of these data and improved data accessibility for both independent experts and MTBs would improve the quality of treatment recommendations. These networks should also provide a harmonized ecosystem for the development of new diagnostic modalities and tools; for example, proteome medicine and AI-based tools.

## 3. Conclusions

Irrespective of the healthcare system, the sustainability and scalability of MTBs are critical to their success and the lack of a standardized process may impact their wide adoption, as well as their efficiency and effectiveness in driving clinical and scientific progress and in improving patient care. Standardization in therapy matching and post-MTB outcomes data collection is also particularly important for MTB value assessment. A properly executed and universal MTB network could allow oncologists to derive benefit from the data collected from patients by MTBs in other countries. The harmonization of practices is therefore critical to the success of MTBs. However, there are several challenges associated with this. National harmonization is hard to accomplish due to the development of parallel structures within a country. For example, academic hospitals have their own local or regional solutions, including diagnostics and MTB standards, and therefore may not strictly adhere to the national or European standards, which need to be clearly defined to help facilitate the convergent development of MTBs. There are also local or regional private or industrial stakeholders, who may offer NGS diagnostics or MTB platforms that may not be translatable to a national or international scale. On the other hand, European harmonization is difficult due to differing resource availability and healthcare structures for patient care, which result in heterogeneous approaches. A lack of recommendations to advance harmonization from scientific societies, and of education or reimbursement for NGS testing and MTB development, may limit the development of broad standards. The implementation of working groups (e.g., similar to the European Scientific foundation for Laboratory Hemato Oncology, which comprises EuroFlow, EuroMRD, and EuroClonality [33]) for the standardization of diagnostics, reporting, and MTB decision making, and for the implementation of these standards at the European level, would help to overcome this hurdle. The ability to adapt these standards at the national level, depending on the healthcare environment, would also help to improve sustainability. Community care commissions and academic hospitals may also need to collaborate with industry to establish standardized diagnostics and reporting. Over 50% of patients across all ages and backgrounds are treated in community practices, which despite offering a sufficient standard of care, may have limited accessibility to study medication. The applicability of MTBs in the community setting is essential for giving these patients equal access to precision oncology. Therefore, it is imperative that these stakeholders are embedded early on in the process of standardization.

Broad NGS panels should be used to test all treatable (ECOG PS 0 or 1) patients in the advanced disease setting, if organ agnostic targeted therapies are locally available. As future data become more complex and the number of patients undergoing genomic profiling increases, MTBs need to be properly set up to deal with these developments and to be able to serve their intended purpose. For MTBs to function effectively in the future, there is a need to establish a national/European network of laboratories with increasing technological complexity, integrated with oncology networks, and including one or more centers capable of performing CGP. In this context, a precise assessment of the need for CGP is essential and could potentially be complemented through collaboration with US Food and Drug Administration (FDA)-approved biotechnology companies for tissue and liquid biopsy molecular profiling. Further to this, epigenetics and proteomics may play a role in precision oncology in the future. Therefore, MTBs should be open to including these new data and incorporating the relevant expertise into their teams, while still being able to utilize the currently available data. Importantly, these data need to be interoperable and sharable in accessible international databases so that collective learning is made possible between MTBs from different countries. Reimbursement is also an issue, as substantial extra work is required for organizing and executing an MTB and this represents a considerable burden on time and resources. This workload should not be delegated to the academic centers but should be implemented in the normal workflow. MTB sustainability will require workforce planning and the education of relevant specialties with regard to the MTB curriculum. Community oncologists may be more open to working with MTBs if they were adequately supported.

Our recommendations described here aim to address these factors and to help MTBs evolve towards integrated, essential components of the oncology healthcare system. Ten key pillars for MTB sustainability and scalability were identified, including suggestions for guidelines for the integration of MTBs into national health systems, where appropriate. A need for scalable frameworks at both the academic center and community-based care levels was also identified. For MTBs in community-based care, this could mean the implementation of on-demand access to oncologists from academic centers, who can join the MTB virtually when needed to help maintain the highest standards in clinical decision making, particularly for patients with rare alterations. Structuring MTBs for guiding precision oncology to achieve the desired patient outcomes is of the utmost importance and requires the involvement of multiple stakeholders. Open dialog should be facilitated between stakeholders, such as government health ministries who are responsible for delivering national precision oncology strategies, research institutions, and European and national regulatory authorities. We hope our recommendations present a call to action to these stakeholders to work together and develop MTBs that can positively impact increasing numbers of patients with cancer now and in the future.

## Data Availability

This manuscript does not report clinical trial results.
